# Prediction of Compressive Strength of Fly Ash-Slag Based Geopolymer Paste Based on Multi-Optimized Artificial Neural Network

**DOI:** 10.3390/ma16031090

**Published:** 2023-01-27

**Authors:** Min Bai, Zhe Zhang, Kaiyue Cao, Hui Li, Cheng He

**Affiliations:** 1 School of Materials Science and Engineering, Chang’an University, Xi’an 710064, China; 2 Engineering Research Central of Pavement Materials, Ministry of Education of P.R. China, Chang’an University, Xi’an 710061, China; 3 School of Materials Science and Engineering, Xi’an Jiaotong University, Xi’an 710049, China

**Keywords:** fly ash and slag, geopolymer paste, compressive strength, artificial neural network

## Abstract

The fly ash-slag geopolymer is regarded as one of the new green cementitious materials that can replace cement, but it is difficult to predict its mechanical properties by conventional methods. Therefore, in the present study, the back propagation (BP) artificial neural network technique is used to predict the compressive strength of the fly ash-slag geopolymer. In this paper, data from the published literature were collected as the training set and the experimental results from laboratory experiments were used as the test set. Eight input parameters were determined, as follows: the percentage of fly ash, the percentage of slag, the water–cement ratio, the curing age, the modulus of alkali activator, the mass ratio of NaOH to Na_2_SiO_3_ and the moles of Na_2_O and SiO_2_ in the alkali activator. Three multilayer artificial neural network models were constructed using the Levenberg–Marquardt (LM), Bayesian regularization (BR) and scaled conjugate gradient (SCG) algorithms to compare the prediction accuracy of the compressive strength of the fly ash-slag geopolymer paste at different ages (3, 7, and 28 d). It was concluded that the training set error of the BR–BP neural network was the smallest. Ultimately, the hyperparameter optimization of the BR–BP neural network was carried out to compare the training set and the test set errors before and after the optimization, and the results show that the BR–BP neural network model with hyperparameter optimization had the highest prediction accuracy.

## 1. Introduction

OPC (ordinary Portland cement) is currently one of the most widely used cementitious materials in the construction industry. However, its production process not only consumes considerable energy, but also emits a significant amount of CO_2_. The cement industry contributes 8% of the world’s greenhouse gas emissions each year [1]. Therefore, the search for a new cementitious material that can replace cement has become a key pursuit in the construction industry.

In 1991, Davidovits [2] prepared a new aluminosilicate inorganic polymer, referred to as a geopolymer, by combining a solid aluminosilicate with an alkali metal silicate solution in an alkaline environment. Over the past three decades, materials such as fly ash, slag, metakaolin, waste glass and biomass ash have been used with an alkali activator to synthesize geopolymers in order to replace cement [3,4,5,6,7,8,9,10,11]. The mechanical properties of geopolymers prepared with pure fly ash were generally poor; however, when the fly ash was partially substituted with slag, the mechanical properties, micro-structure and erosion resistance of the geopolymers were significantly enhanced [12,13,14,15,16]. Consequently, an investigation into the properties of a fly ash and slag geopolymer became the research objective of the present study. However, the reaction mechanisms of cement-based materials cannot be used to directly explain the geopolymer, due to differences between the polymerization reaction of geopolymers and the reaction mechanisms of cement-based materials. The performance of geopolymers varies significantly under the influence of different factors such as the type of fly ash, different alkaline activators and the different processes for the preparation of geopolymers, which can result in a high variability of the mix proportions of the geopolymer materials.

The traditional mathematical analysis methods for calculating and predicting the compressive strength of the geopolymer paste can lead to complicated calculations, which are time-consuming and provide poor predictability [17]. The artificial neural network algorithm can theoretically approximate any function. The basic structure of a neuron is composed of nonlinear change units and there is a strong nonlinear mapping ability in the neural network which means that better prediction results can be achieved for complex nonlinear problems by utilizing the artificial neural network. In addition, parameters such as the number of intermediate layers of the network, the number of processing units in each layer and the learning rate of the network are very flexible and can be set according to the specific situation. Therefore, the artificial neural network has widespread application prospects in many fields; for example, optimization [18], signal processing [19], pattern recognition [20], intelligent control [21], and fault diagnosis [22].

The artificial neural network is an algorithmic model that simulates biological neural behavior for distributed parallel information processing, with the capacity for self-learning and self-adaptation. In 1986, Rumelhart, McClelland [23] and other scientists first proposed the idea of the back propagation (BP) neural network. In comparison with the conventional artificial neural network algorithm, its primary characteristic is the addition of the error back propagation to signal the forward transmission. In this kind of artificial neural network, it will revert to back propagation if the output layer cannot achieve the expected output. The weights and biases in the network are adjusted based on the prediction error to guarantee that the prediction output of the BP neural network can approach the expected output constantly and achieve the effect of model optimization.

However, there are still some issues with the standard BP neural network. For instance, the weights and biases are generated at random before each training, arriving at different results for each program running. Therefore, multiple simulations are required to select the best weights and biases, although there is no assurance that the weights and biases will be the best. Therefore, the initial neural network requires optimization. There are three primary methods for neural network model optimization. The first method uses different intelligent algorithms to optimize the standard BP neural network. The second method is to debug and optimize the number of hidden layers and the neural nodes of hidden layers. The last method is to optimize the hyperparameter, including the weights, biases, training times, learning rates, target errors, etc.

The hyperparameter optimization optimizes the neural network by adjusting the hyperparameters, which debug the established neural network from the source code. It is a purposeful operation compared with the randomness of the results of the first two methods, and the debugging effect of this last method is generally better than the first two algorithms. Although the advantages of this approach are evident, it requires skillful computer programming and sets advanced requirements for the material sciences considering that so few articles on the hyperparameter optimization of neural networks have been published in this field. 

Recent research on the single-layer artificial neural network has focused on the neural network modeling of geopolymer concrete [24,25,26,27,28,29,30,31,32,33,34,35], while fewer studies have addressed neural network modeling for geopolymer pastes. An additional problem involves the generalizability of the model. Some studies incorporate only their own data to construct and validate the models, yet ignore the models’ generalizability. Although some papers cite data from the literature to support the construction of the models and validate them with their own experimental data, the prediction results of these models are often poor. This is because the structure of the single-layer neural network is too simple or is not trained by hyperparameter optimization; therefore, this results in either the underfitting (where the prediction result deviates significantly) or the overfitting (where the model falls into local optimization) of the model, which reflects the reduced reliability of the fit of the neural network model. With an increase in the number of hidden layers and neuron nodes, the more complex multilayer neural networks can effectively remedy the problems of single-layer neural networks, due to their exponential growth in the degree of calculation in the process of model-building and verification.

In this paper, the multilayer neural network technique was achieved through MATLAB software coding. The complexity of the multilayer neural network technique requires a large and complex number of modeling codes, which are used in this paper and include three different algorithms, the optimum number of nodes in the different hidden layers and the hyperparameter optimization codes all written by the authors themselves and implemented by the MATLAB decoder, instead of using the built-in toolbox of MATLAB (the built-in toolbox only enables the modeling of single-layer neural networks). A multilayer BP neural network model was constructed for predicting the compressive strength of fly ash and slag geopolymer paste at different ages. The number of node structures’ optimization, the algorithm comparison optimization and the hyperparameter optimization were carried out sequentially to ensure the reliability and the accuracy of the multilayer neural network model, which was established through multi-path and multi-modality training over multiple times.

## 2. Experiments

### 2.1. Materials

The Class F type of fly ash was used in this study. Both the fly ash and slag were from the Hancheng Power Plant of Shaanxi Datang Power Co., Ltd., and their chemical composition was analyzed by an X-ray fluorescence analyzer, as shown in Table 1.

NaOH is a commercially available industrial solid soda ash with a purity of 99%. NaOH pellets were dissolved in water to prepare a solution, which was stirred for at least 10 min to ensure all the pellets were dissolved. After cooling, the NaOH solution was mixed thoroughly with the Na_2_SiO_3_ in prescribed proportions to prepare the alkaline activator. The chemical composition and physical properties of the liquid, Na_2_SiO_3_, according to the standard GB/T 4209-2008, are listed in Table 2. 

### 2.2. Experimental Design

The mechanical strength of a geopolymer is affected by various factors, including the type of alkali activator [36], the content [37,38] and the modulus of the alkali activator [39,40], the type and content of cementitious materials [41,42], the curing conditions [43,44], etc. In the present study, the raw materials for the preparation of the geopolymer were used as variables according to a three-factor and four-level test design. The water–binder ratio was controlled to 0.27 for all the experimental groups. Table 3 illustrates the mixing process for the geopolymer. The three factors were: the ratio of fly ash to slag, the activator’s content and the modulus of the alkali activator. Each factor had four levels, described as follows. For the ratio of fly ash to slag, the level distribution was 85/15, 80/20, 75/25 and 70/30, for the four levels, respectively. The four levels of the activator content were 10, 15, 20 and 25% of the binding material, and the four levels of the modulus of the water glass were 0.5, 1.0, 1.5 and 2.0.

### 2.3. The Test Procedure

The fly ash and slag were weighed and dry-mixed for 3 minutes, after which the alkali activator was added with slow stirring for 2 minutes and fast stirring for 5 minutes. Subsequently, the mixture was vibrated for 2 minutes and 60 seconds on the cement sand vibration table. The specimens were prepared according to the Chinese standard GB/T 17671-2021 with a size of 40 × 40 × 160 mm. All specimens were stored at 23 ± 2 °C and ≥95% relative humidity for 24 h before being demolded and cured at the same temperature and relative humidity. When the specimens reached 3, 7, and 28 d, respectively, the compressive strength was evaluated. The compressive strength was tested by using a universal testing machine with a maximum capacity of 3000 kN. The loading rate was set at 2.4 kN/s.

## 3. Analysis of Test Results

**Figure 1:**
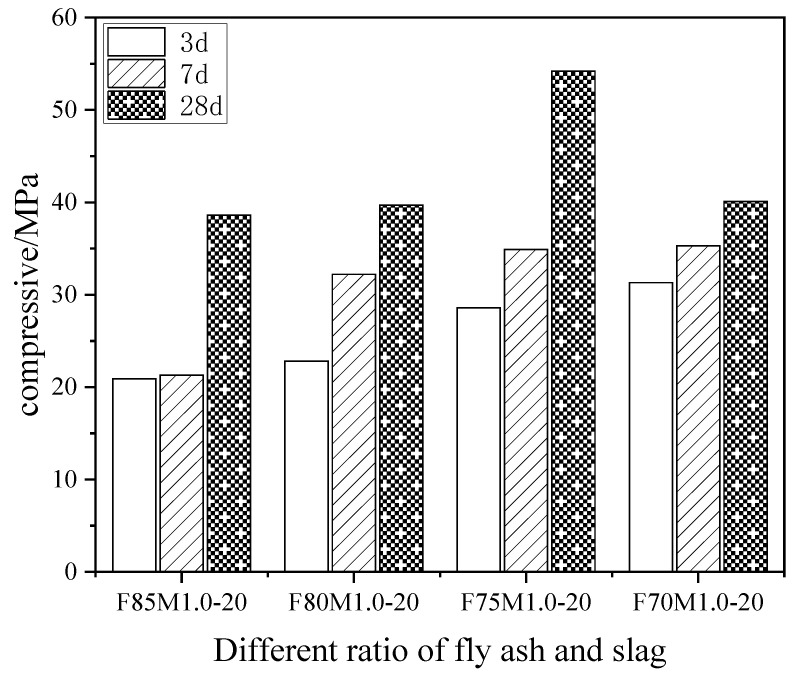
The effects of different fly ash content at different ages on the compressive strength of geopolymers.

Figure 1 illustrates the effects of different fly ash contents at different ages on the compressive strength of the geopolymers with the modulus of the alkali activator at 1.0 and the content of alkali activator at 20%. When the quantity of fly ash remained constant, the compressive strength of the geopolymers was positively correlated with the curing age. At 3 and 7 d of curing, the compressive strength of the geopolymers increased as the fly ash content decreased, but this trend seemed to level out gradually. After 28 days of curing, the compressive strength of the geopolymers increased initially, then decreased with the declining fly ash content. The maximum compressive strength of the geopolymers was achieved when the fly ash comprised 75% of the total mass of the binding material. The increase in slag content, as well as the stability in the alkali activator admixture and the alkali activator modulus, demonstrated that through the preliminary geological polymerization reaction the high CaO content in the slag supported the C-S-H gel formation in conjunction with a stable silica-aluminate structure. The compressive strength of the geopolymer paste tended to increase with the increase in the slag admixture. However, the increasing amount of slag also accelerated the hydration reaction rate of the geopolymer paste and shortened the setting and hardening time. The accelerated hydration reaction rate led to a greater number of microcracks in the hardened geopolymer paste, which negatively impacted the compressive strength of the geopolymer paste; therefore, the compressive strength of the geopolymer paste decreased after the slag substitution amount exceeded 25%.

**Figure 2:**
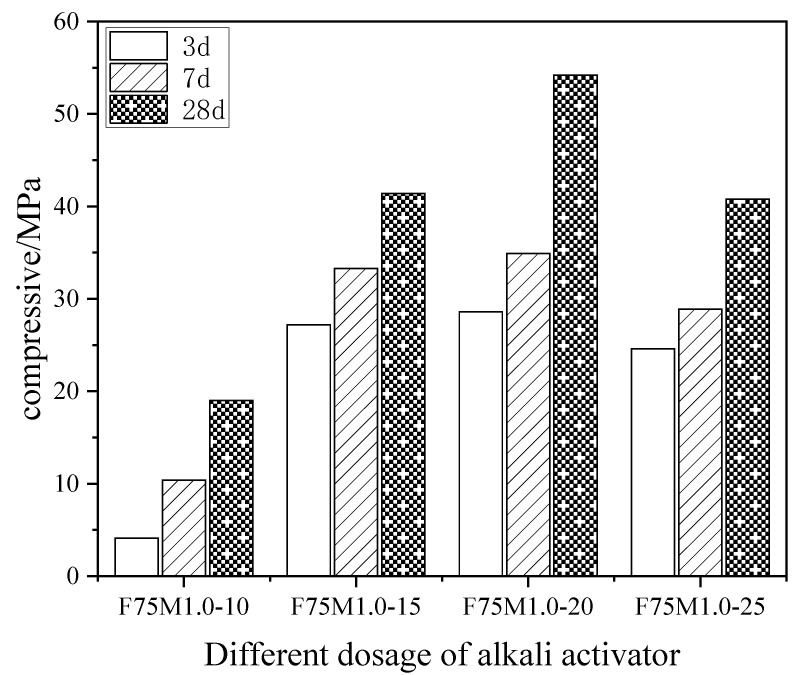
The effects of different alkali activators content on the compressive strength of the geopolymer.

Figure 2 depicts the effects of the different alkali activator contents on the compressive strength of the geopolymer at various ages when the ratio of fly ash to slag and the alkali activator modulus were 75/25 and 1.0, respectively. A constant amount of alkali activator achieved an increase in the compressive strength of the geopolymers as the maintenance age increased. An increasing content of alkali activator caused the compressive strength of the geopolymers to increase and then decrease at the same age. However, when the alkali activator was 20%, the compressive strength of the geopolymer reached its maximum. The OH^−^ concentration in the system increased gradually with the increase in the alkali activator content, when the ratio of fly ash to slag and the modulus of the alkali activator were stable. The increase in alkali concentration caused an acceleration in the dissolution rate of the silica-alumina monomer, an acceleration in the polymerization reaction rate, the generation of more hydration products and an improvement in the mechanical properties of the geopolymer. An excessive alkali concentration prevented the polycondensation reaction and reduced the generation of the Si-O-Al polymer. In addition, an excessive alkali concentration restricted the diffusion of ions and delayed the formation of the polymer, which reduced the compressive strength of the geopolymer.

**Figure 3:**
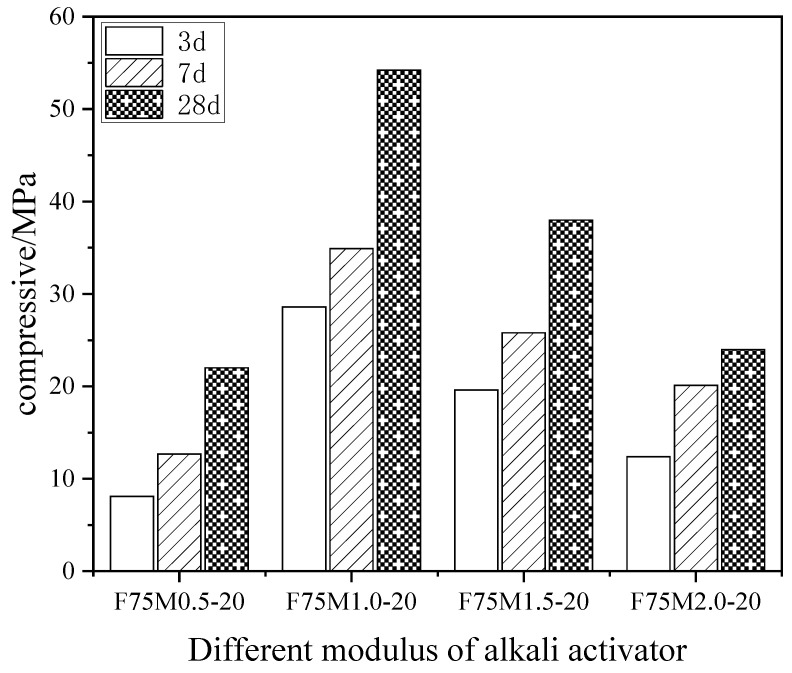
The effect of the different alkali activators modulus on the compressive strength of the geopolymer.

Figure 3 shows the effect of the different alkali activators modulus on the compressive strength of the geopolymer at the different ages when the ratio of fly ash to slag was 75/25 and the alkali activator dosage was 20%. The compressive strength of the geopolymers increased with the curing age when the modulus of the alkali activator was identical. The compressive strength of the geopolymers initially increased and then decreased at the same age, as the modulus of the alkali activator increased. The compressive strength of the geopolymer reached its maximum when the modulus of the alkali activator was 1.0. This increase in the alkali activator modulus indicated that the system contained a higher level of SiO_2_, while the proportion of fly ash to slag and the amount of alkali activator remained stable. The increased SiO_2_ enhanced its reaction with CaO to form more C-S-H gels and Ca-rich geopolymer gels. An alkali activator modulus of greater than 1.0 indicated that the alkalinity in the solution was insufficient and the polymerization reaction was poor. Furthermore, the degree of silicate polymerization in the solution increased and the structure of the Na_2_O-nSiO_2_ was more stable because the modulus of the alkali activator was too large, which resulted in polycondensation in the polymerization process and a decrease in the strength of the geopolymer.

## 4. BP Neural Network

A single hidden layer network in neural networks can fit a function of “continuous mapping from one finite space to another”. A double hidden layer network with appropriate activation functions can represent any decision boundary with arbitrary precision and can fit any smooth mapping with precision, and a triple or higher hidden layer network can learn more complex descriptions. 

In most circumstances, a neural network with a single hidden layer is sufficient [45,46]. However, because the response of the geopolymers is more complex, in order to exclude the chance of fitting results with a single hidden layer, as well as to improve the performance and accuracy of the network, a neural network with two hidden layers is established in this paper. In the neural network model, there is no explicit rule for the number of nodes of neurons, and the default value of 5 or 10, or any value determined by the authors, is usually adopted. In order to determine the optimal number of hidden neuron nodes, two empirical formulas (Equation (1) and Equation (2)), which are well recognized in the field of deep learning for determining the number of hidden layers neuron nodes, are cited in this paper as the basis for determining the number of neuron nodes in the first and second layers, respectively:

(1) $$N=\sqrt{m+n}+\alpha$$

where m represents the number of input layer nodes, N represents the number of hidden neuron nodes, n represents the number of output layer nodes and α represents a number from 1 to 10.

(2) $$N=\frac{Ns}{\beta *\left(m+n\right)}$$

where β is a constant between 2 and 10 and Ns denotes the quantity of model samples.

In this article, to assess the efficiency of the neural network model fitting, the mean square error (MSE), mean absolute error (MAE) and the correlation coefficient (R) are utilized. The MSE and MAE are calculated using Equations (3) and (4).

(3) $$MSE=\frac{1}{m}\sum_{i=1}^{m}{\left(y_{i}-\hat{y_{i}}\right)}^{2}$$

(4) $$MAE=\frac{1}{n}\sum_{i=1}^{n}\left|\hat{y_{i}}-y_{i}\right|$$

Notably, the initial weights and biases of the neural network are generated randomly, resulting in different training outcomes each time. By modifying the underlying code, this paper ensures that the initial weights and biases are the same in each run. The optimal number of hidden layer neuron nodes is found by comparing the mean square error (MSE) of each resulting model and by fixing the initial weights and thresholds and changing the number of neuron nodes in each hidden layer.

In order to ensure the generalizability of the model, 158 data sets were chosen from references and experimental results [47,48,49,50]. There were eight input parameters: the percentage of fly ash, the percentage of slag, the water–cement ratio, the curing age, the modulus of alkali activator, the mass ratio of NaOH to Na_2_SiO_3_, and the moles of Na_2_O and SiO_2_ in the alkali activator. The parameter output was compressive strength. Table 4 displays the parameters of the neural network.

An appropriate algorithm must be selected to train a neural network. The Levenberg–Marquardt, Bayesian regularization and scaled conjugate gradient are the most developed and widely utilized theoretical frameworks for neural network training.

### 4.1. Collection and Processing of Data

Before performing the data network modeling, the input data must be normalized. The study conducted by M. Aminul Haque et al. [51] demonstrated that the difference between the errors in the resulting neural networks, before and after comparing the data for normalization, was large. In this study, a commonly used linear normalization expression was used to scale the input data points for training and testing to a range from −1 to 1 according to Equation (5): where X represents the normalized data; X_i_ represents the input data; and $X_{max}$ and $X_{min}$ represent the minimum and maximum values of the input data, respectively.

(5) $$X=\frac{X_{i}-X_{min}}{X_{max}-X_{min}}$$

After the modeling is completed, it is generally necessary to also perform the inverse normalization process, i.e., to invert the true value by this formula.

### 4.2. The Levenberg–Marquardt Artificial Neural Network 

**Figure 4:**
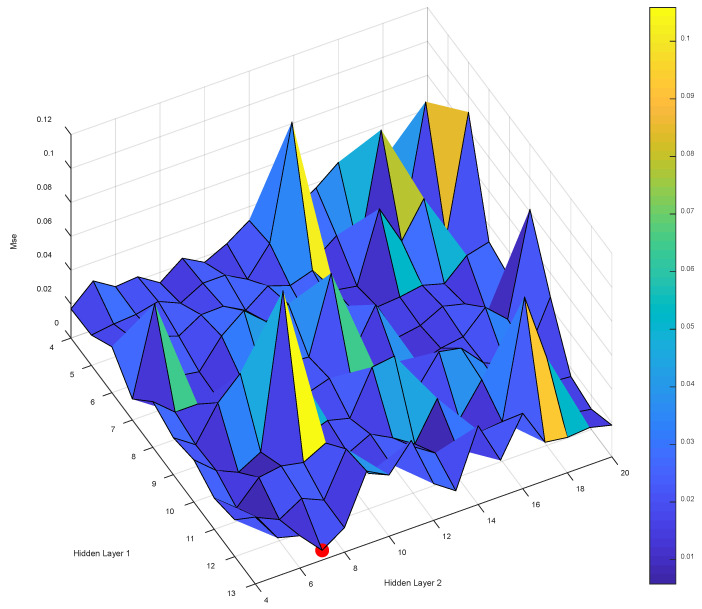
The effect of the hidden layer nodes on the training error of LM-ANN.

After determining the range of values for the number of nodes in the first and second hidden layers according to the empirical formula, the neural network modeling is performed, and the MSE of all models built by the LM-ANN can be obtained when the number of neuron nodes in the first and second hidden layers are different, as shown in Figure 4. (Detailed information is provided in attached Table 1.)

The number of neuron nodes in the first hidden layer and second hidden layer were used as the X and Y axes, respectively. The evaluation index MSE was used as the Z axis. When the established model has the smallest MSE, the best performance of the established model is achieved. After we built 170 models, we obtained the minimum MSE value of 0.0057733 when the number of nodes in the first hidden layer was 13 and the number of nodes in the second hidden layer was seven. Table 5 lists the technical specifications of the LM-ANN.

**Figure 5:**
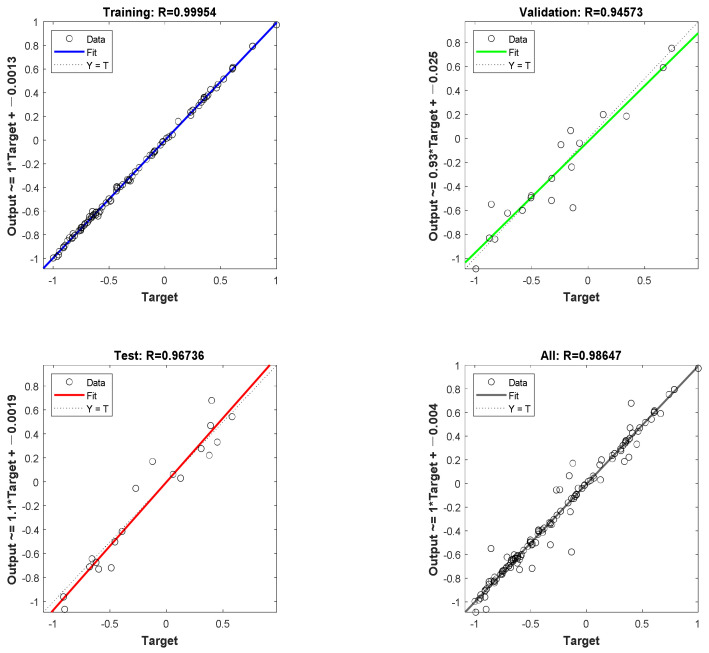
LM-ANN model fitting.

The final result of the LM-ANN model fitting is shown in Figure 5. The LM-ANN had a strong match with R being equal to 0.99954 for the training set, R being equal to 0.94573 for the validation set, R being equal to 0.96736 for the test set, and R being equal to 0.98647 for the overall model. Table 6 displays the evaluation indicators for each of the actual values calculated by Equations (3) and (4) after the final LM-ANNS model was subjected to the inverse normalization operation.

### 4.3. The Bayesian Regularization Artificial Neural Network 

**Figure 6:**
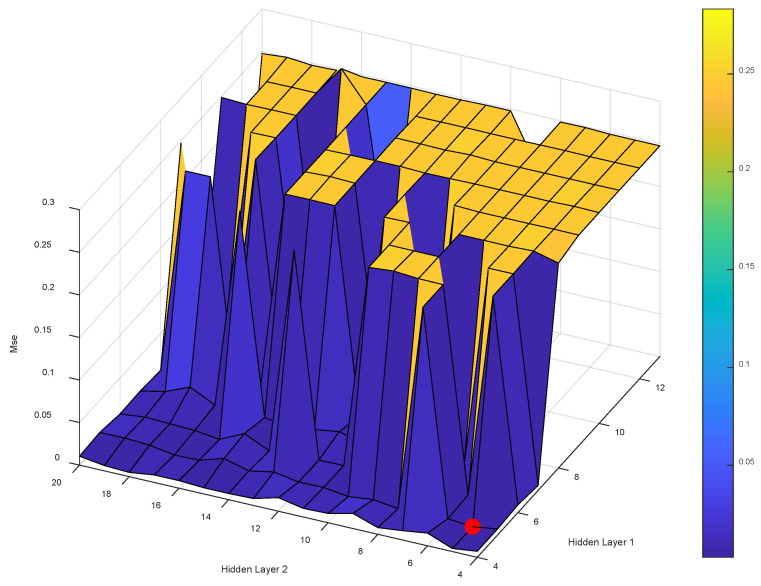
The effect of the hidden layer nodes on the training error of BR-ANN.

As shown in Figure 6, the MSE of the BR-ANN model was built by traversing the number of neuron nodes in the first hidden layer and the number of neuron nodes in the second hidden layer (see attached Table 2 for detailed data). After creating 170 models using the BR algorithm, we concluded that when the number of neural nodes in the first hidden layer was five and the number of neural nodes in the second hidden layer was five, the smallest MSE value was 0.002947. The technical parameters of the neural network of the BR-ANN are consistent with the LM-ANN.

**Figure 7:**
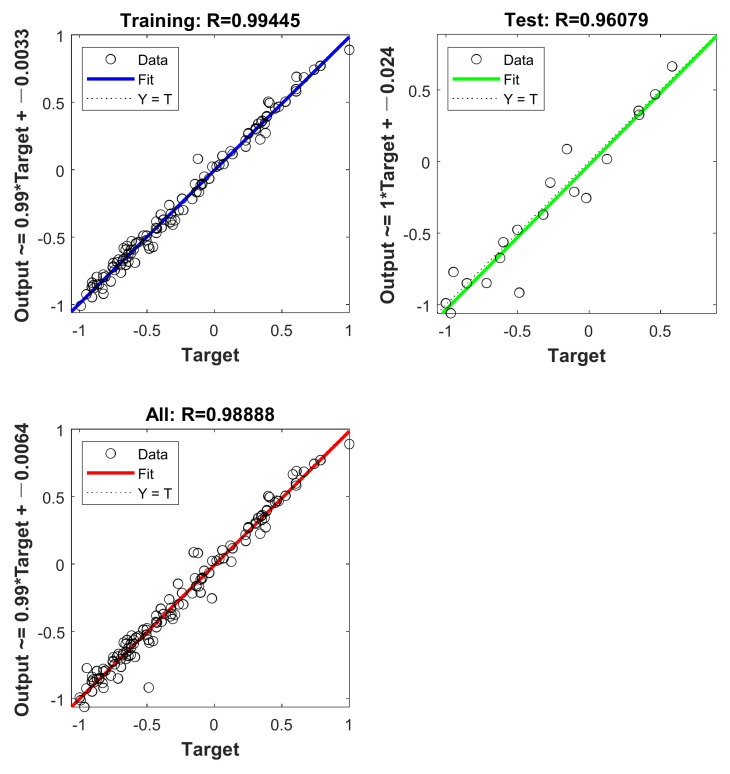
BR-ANN model fitting.

Figure 7 depicts the final effect of the BR-ANN model fitting. From Figure 7, it can be seen that the training set, the test set and the overall model R are equal to 0.99445, 0.96079 and 0.98888, respectively. Table 7 shows the evaluation indexes of the actual values obtained after the inverse normalization operation of the final BR-ANN model.

### 4.4. The Scaled Conjugate Gradient Artificial Neural Network

**Figure 8:**
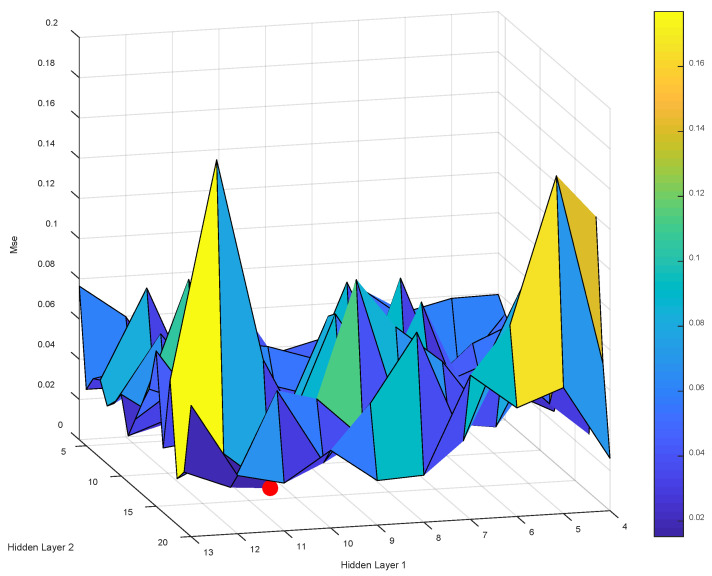
The effect of the hidden layer nodes on the training error of SCG-ANN.

In Figure 8, the MSE of the SCG-ANN model was built by traversing the number of neuron nodes in the first hidden layer and the number of neuron nodes in the second hidden layer (see attached Table 3 for detailed data). After building 170 models using the SCG algorithm, we concluded that when the number of neural nodes in the first hidden layer was 11 and the number of neural nodes in the second hidden layer was 18, the smallest MSE value was 0.015232. The technical parameters of the neural network of the SCG-ANN are in line with BR-ANN and LM-ANN.

**Figure 9:**
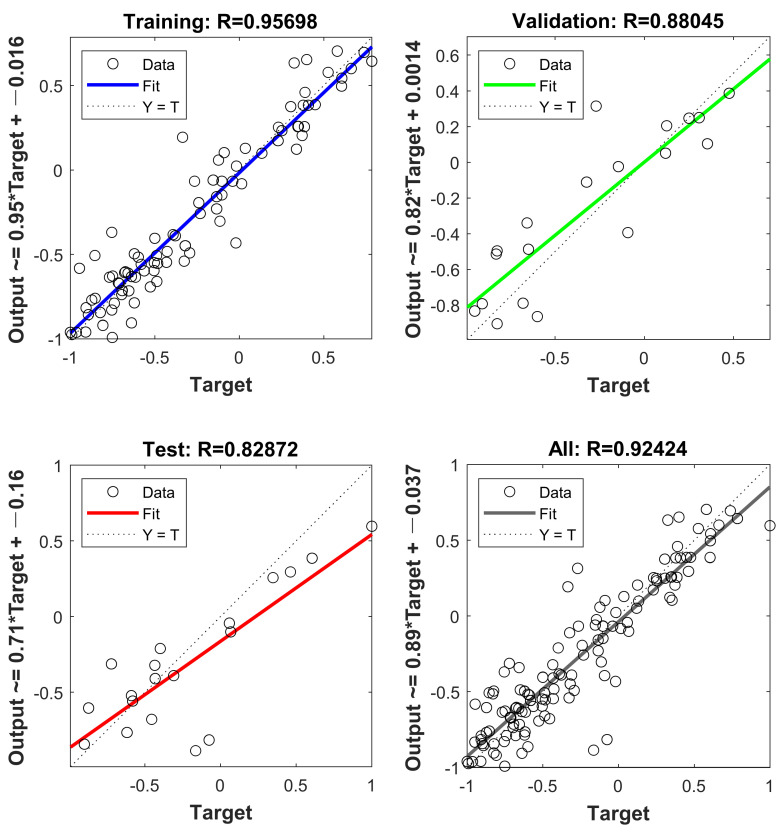
SCG-ANN model fitting.

Figure 9 depicts the final effect of the SCG-ANN model fitting, showing that the training set, the validation set, the test set and the overall model R are equal to 0.95698, 0.88045, 0.82872 and 0.92424 respectively. Table 8 shows the evaluation metrics for each of the actual values obtained after the final SCG-ANN model was subjected to the inverse normalization operation.

A comparison of the three different algorithms and the number of neuron nodes in each hidden layer reveals that the model trained with the BR algorithm had the best training set fit, but that the test set was inadequately tested. In order to increase the adequacy of the test set, we used hyperparameter optimization to further optimize the model in this paper.

### 4.5. Hyperparameter BR Model Optimization

The technical details of hyperparameter optimization include two aspects: (i) the implementation of debugging changes to the basic technical parameters of the neural network, including the times of training iterations, minimum training gradient, maximum momentum, learning rate, minimum training target, and the maximum number of failures. (ii) Debugging the weights and biases between two hidden layers, and the weights and biases between the hidden layers and output layer. The technical parameters of the BR-ANN neural network following hyperparameter optimization are displayed in Table 9.

Based on the three hyperparameters (Number of iterations, Training gradient and Learning rate) of the previous BR-ANN, the three hyperparameters were debug several times, and the debugging was stopped when the training effect could not be further optimized. The target goodness of fit was set to 1. To prevent the neural network from falling into an infinite loop, the maximum number of training cycles was limited to 50,000, and the set of models with the largest goodness of fit value was selected as the final output model. To facilitate the distinction, the BR-ANN, before optimization, was referred to as P-BR, and the BR-ANN after hyperparameter optimization was referred to as AO-BR.

**Figure 10:**
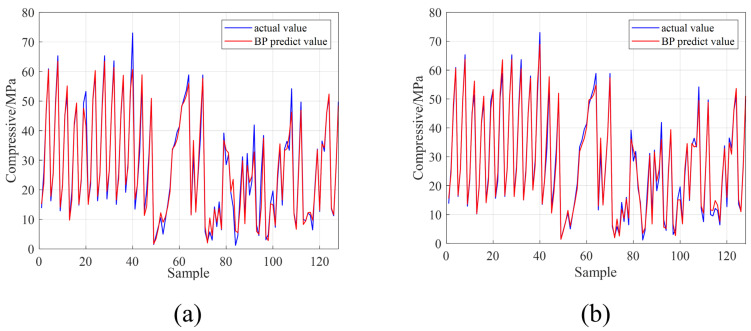
The actual value and the predict value of the training sets (**a**) the P-BR and (**b**) the AO-BR.

Figure 10a,b shows the true values and model predictions in the P-BR and AO-BR training sets. The error between the true and predicted values in the training set was used to indicate the goodness of the model: when the higher degree of overlap between the true and the predicted data represented the smaller error between the true and the predicted values, the model of the neural network was better. Figure 10 shows that, after hyperparameter optimization, the degree of overlap between the true and the predicted values of the training set data further increased. Table 10 shows the three evaluations metrics for the P-BR and AO-BR training sets. Table 10 shows that, after hyperparameter optimization, although the MSE and MAE changed from 7.1374 and 1.7552 to 9.3144 and 2.0017, respectively, with a small increase, the final R-value increased from 0.9889 to 0.9924, indicating that the final impact of hyperparameter optimization on the accuracy of the model training set was positive. When we combine Figure 10 and Table 10, it is evident that after hyperparameter optimization, the training set of the neural network model achieves a better fitting effect.

**Figure 11:**
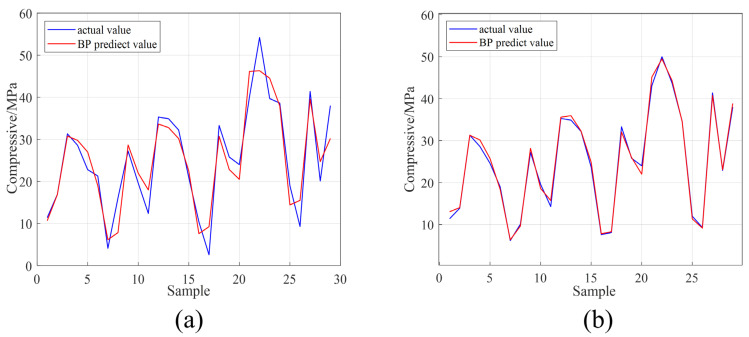
The actual value and the predict value of the test sets (**a**) the P-BR and (**b**) the AO-BR.

Figure 11a,b shows the true values and the model prediction values in the P-BR and AO-BR test sets. The error between the true and predicted values in the test set can indicate the successful or unsuccessful generalizability of the model, and when the error between the true and predicted values is smaller, it proves that the prediction performance of the neural network is more accurate. Figure 11 shows that, before hyperparameter optimization, the difference between the true value and the predicted value given by the neural network was large, which proves that the prediction ability of the neural network was poor. After hyperparameter optimization, the difference between the true value and the predicted value was very small, which demonstrates that the prediction ability of the neural network was extremely good after hyperparameter optimization. Table 11 shows the three evaluations metrics for the P-BR and AO-BR test sets. From Table 11, it is evident that the MSE and MAE of the test set were improved by five and three times, respectively. The R-value improved significantly from 0.6233 to 0.9408, suggesting that for this model, the optimization is highly accurate. Combining Figure 11 and Table 11 shows that the generalizability of the test set of the neural network model has been greatly improved after hyperparameter optimization.

**Figure 12:**
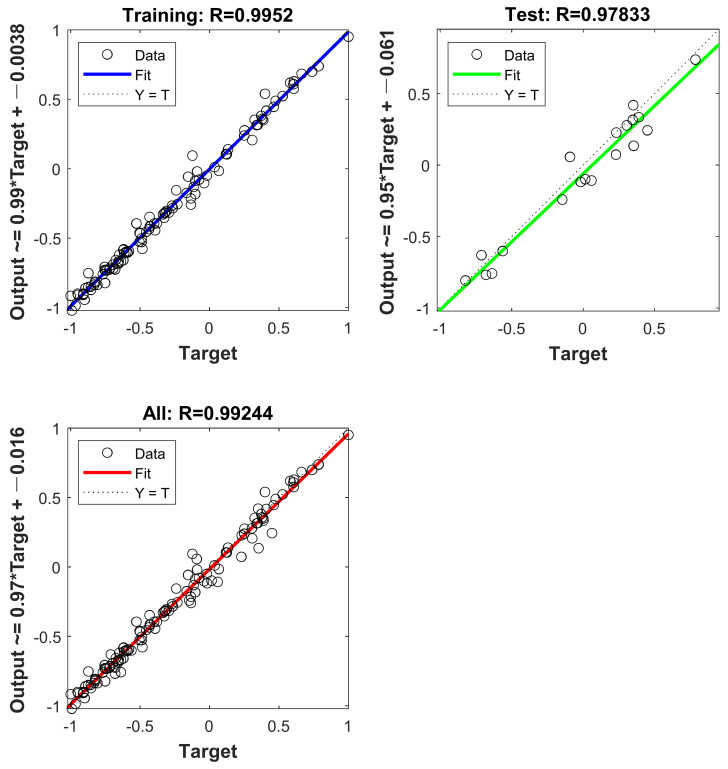
AO-BR model fitting.

The AO-BR model is depicted in Figure 12. The goodness of fit of the BR-ANN was R = 0.9952 for the training set, R = 0.97833 for the test set and R = 0.99244 for the overall model, as shown in Figure 12.

## 5. Conclusions

This study aimed to predict the compressive strength of fly ash-slag geopolymer paste using artificial neural networks. A database of artificial neural network models was constructed from the published literature data, and the accuracy of the artificial neural network model predictions was verified by the results of laboratory tests. The following conclusions were obtained:

(1). The percentage of fly ash, the percentage of slag, the water–cement ratio, the curing age, the modulus of the alkali activator, the mass ratio of NaOH to Na_2_SiO_3_, and the moles of Na_2_O and SiO_2_ in the alkali activator are used as input parameters of the artificial neural network, and R, MSE and MAE are used as evaluation indexes of the prediction accuracy of the artificial neural network.
(2). The optimal structures of multilayer neural networks constructed by different algorithms are not the same. When the number of nodes in the two hidden layers of the LM algorithm is 13 and 7, respectively, the LM-ANN has the minimum errors of MSE = 8.7616, MAE = 1.435, and R = 0.9565. When the number of nodes in the two hidden layers of the BR algorithm is 5 and 5, respectively, the BR-ANN has the minimum errors of MSE = 7.1374, MAE = 1.7552 and R = 0.9889. The SCG-ANN has the smallest error when the number of nodes of the two hidden layers of the SCG algorithm is 11 and 18, respectively, with the evaluation metrics of MSE = 47.0747, MAE = 4.8827, and R = 0.9242. This indicates that the model accuracy of the BR-ANN is better than the LM-ANN and the SCG-ANN.
(3). Using the hyperparameter optimization technique for the BR-ANN, the test set errors of the P-BR are MSE = 156.5307, MAE = 10.4124 and R = 0.6233; the test set errors of the AO-BR are MSE = 17.1564, MAE = 3.415 and R = 0.9408. This indicates that the artificial neural network has better generalizability after hyperparametric optimization.
(4). The results of this study show that by relying on the high prediction accuracy of artificial neural network technology, the mechanical properties of geopolymer materials at the target age can be predicted in advance based on the design ratios of the geopolymer materials. Artificial neural networks are also more financially viable and time-efficient when we consider their application for geopolymer materials in laboratory studies and actual architectural construction, and thus are beneficial for promoting the further development of geopolymer materials.

## Figures and Tables

**Table 1:** Chemical composition of fly ash and slag.

	Chemical Composition	CaO	Al_2_O_3_	SiO_2_	Fe_2_O_3_	MgO	TiO_2_	MnO	Na_2_O	K_2_O	P_2_O_5_	Loss
Fly-ash	content/%	5.33	28.10	51.50	6.58	0.83	1.10	0.83	0.49	1.55	0.18	3.73
Slag	content/%	34.95	17.26	32.90	0.89	8.04	1.18	8.04	0.33	0.50	0.04	1.89

**Table 2:** Chemical composition and physical properties of water glass.

Product Name	SiO_2_/%	Na_2_O/%	Density Be/20 °C	Modulus
SP50	29.99	13.75	50Be	2.25

**Table 3:** Mixture proportion of geopolymer.

Test Group Number	Specimens ID	Fly Ash/Slag/wt%	Alkali Activators Modulus	Alkali Activators Dosage/%
1	F85M1.0–20	85/15	1.0	20
2	F80M1.0–20	80/20	1.0	20
3	F75M1.0–20	75/25	1.0	20
4	F70M1.0–20	70/30	1.0	20
5	F75M1.0–10	75/25	1.0	10
6	F75M1.0–15	75/25	1.0	15
7	F75M1.0–20	75/25	1.0	20
8	F75M1.0–25	75/25	1.0	25
9	F75M0.5–20	75/25	0.5	20
10	F75M1.0–20	75/25	1.0	20
11	F75M1.5–20	75/25	1.5	20
12	F75M2.0–20	75/25	2.0	20

The meaning of symbols in the Specimens ID: F represents fly ash. The modulus of the alkali activators is denoted by M. The number following “-” indicates the quantity of alkali activators. “F85M1.0–20” indicates that the binder comprised 85% fly ash, the modulus of alkali activators is 1.0 and the mass of alkali activators is equal to 20% of the binder mass.

**Table 4:** The values of the neural network’s parameters.

Variable		Parameters Range	Code	Category
Fly ash (wt%)		0–100	X1	Input
Slag (wt%)		0–50	X2	
Water–cement ratio		0.27–0.44	X3	
Curing age (day)		3/7/28	X4	
NaOH:Na_2_SiO_3_ (wt%)		0.0222–0.6211	X5	
Alkali activator	modulus (M)	0.2–2	X6	
	Na_2_O (mol)	0.18–1.76	X7	
	SiO_2_ (mol)	0.18–1.56	X8	
Compressive strength (MPa)		5.60–105.46	Y	output

**Table 5:** LM-ANN’s technical parameters.

Details	Selection
Number of inputs	8
Number of hidden layers	2
Number of outputs	1
Number of neurons in hidden layer 1	13
Number of neurons in hidden layer 2	7
Number of iterations	1000
Training gradient	1 × 10^−5^
Learning rate	0.1
Validation checks	6
Activation function	TanSig and LogSig

**Table 6:** Evaluation metrics for training set and test set.

	MSE	MAE	R
Training set	8.7616	1.435	0.9565
Test set	109.7549	8.1861	0.7781

**Table 7:** Evaluation metrics for training set and test set.

	MSE	MAE	R
Training set	7.1374	1.7552	0.9889
Test set	156.5307	10.4124	0.6233

**Table 8:** Evaluation metrics for training set and test set.

	MSE	MAE	R
Training set	47.0747	4.8827	0.9242
Test set	80.6257	7.0759	0.8165

**Table 9:** The technical parameters of the BR-ANN.

Details	Selection
Number of inputs	8
Number of hidden layers	2
Number of outputs	1
Number of neurons in hidden layer 1	5
Number of neurons in hidden layer 2	5
Number of iterations	50,000
Training gradient	1 × 10^−5^
Learning rate	1 × 10^−4^
Validation checks	6
Activation function	TanSig and LogSig

**Table 10:** Evaluation metrics for training set of the P-BR and the AO-BR.

	MSE	MAE	R
P-BR	7.1374	1.7552	0.9889
AO-BR	9.3144	2.0017	0.9924

**Table 11:** Evaluation metrics for test set of the P-BR and the AO-BR.

	MSE	MAE	R
P-BR	156.5307	10.4124	0.6233
AO-BR	17.1564	3.415	0.9408

## Data Availability

Not applicable.
